# Classification of bioinformatics workflows using weighted versions of partitioning and hierarchical clustering algorithms

**DOI:** 10.1186/s12859-015-0508-1

**Published:** 2015-03-03

**Authors:** Etienne Lord, Abdoulaye Baniré Diallo, Vladimir Makarenkov

**Affiliations:** 1 Département d’informatique, Université du Québec à Montréal, C.P. 8888 succ. Centre-Ville, Montreal, QC H3C 3P8 Canada; 2 Département de sciences biologiques, Université à Montréal, C.P. 6128 succ. Centre-Ville, Montreal, QC H3C 3J7 Canada

**Keywords:** Bioinformatics workflows, Hierarchical clustering, *k*-means partitioning, Scientific workflows, Workflow clustering

## Abstract

**Background:**

Workflows, or computational pipelines, consisting of collections of multiple linked tasks are becoming more and more popular in many scientific fields, including computational biology. For example, simulation studies, which are now a must for statistical validation of new bioinformatics methods and software, are frequently carried out using the available workflow platforms. Workflows are typically organized to minimize the total execution time and to maximize the efficiency of the included operations. Clustering algorithms can be applied either for regrouping similar workflows for their simultaneous execution on a server, or for dispatching some lengthy workflows to different servers, or for classifying the available workflows with a view to performing a specific keyword search.

**Results:**

In this study, we consider four different workflow encoding and clustering schemes which are representative for bioinformatics projects. Some of them allow for clustering workflows with similar topological features, while the others regroup workflows according to their specific attributes (*e.g.* associated keywords) or execution time. The four types of workflow encoding examined in this study were compared using the weighted versions of *k*-means and *k*-medoids partitioning algorithms. The Calinski-Harabasz, Silhouette and logSS clustering indices were considered. Hierarchical classification methods, including the UPGMA, Neighbor Joining, Fitch and Kitsch algorithms, were also applied to classify bioinformatics workflows. Moreover, a novel pairwise measure of clustering solution stability, which can be computed in situations when a series of independent program runs is carried out, was introduced.

**Conclusions:**

Our findings based on the analysis of 220 real-life bioinformatics workflows suggest that the weighted clustering models based on keywords information or tasks execution times provide the most appropriate clustering solutions. Using datasets generated by the *Armadillo* and *Taverna* scientific workflow management system, we found that the weighted cosine distance in association with the *k-*medoids partitioning algorithm and the presence-absence workflow encoding provided the highest values of the Rand index among all compared clustering strategies. The introduced clustering stability indices, PS and PSG, can be effectively used to identify elements with a low clustering support.

**Electronic supplementary material:**

The online version of this article (doi:10.1186/s12859-015-0508-1) contains supplementary material, which is available to authorized users.

## Background

### Introduction

**Figure 1:**
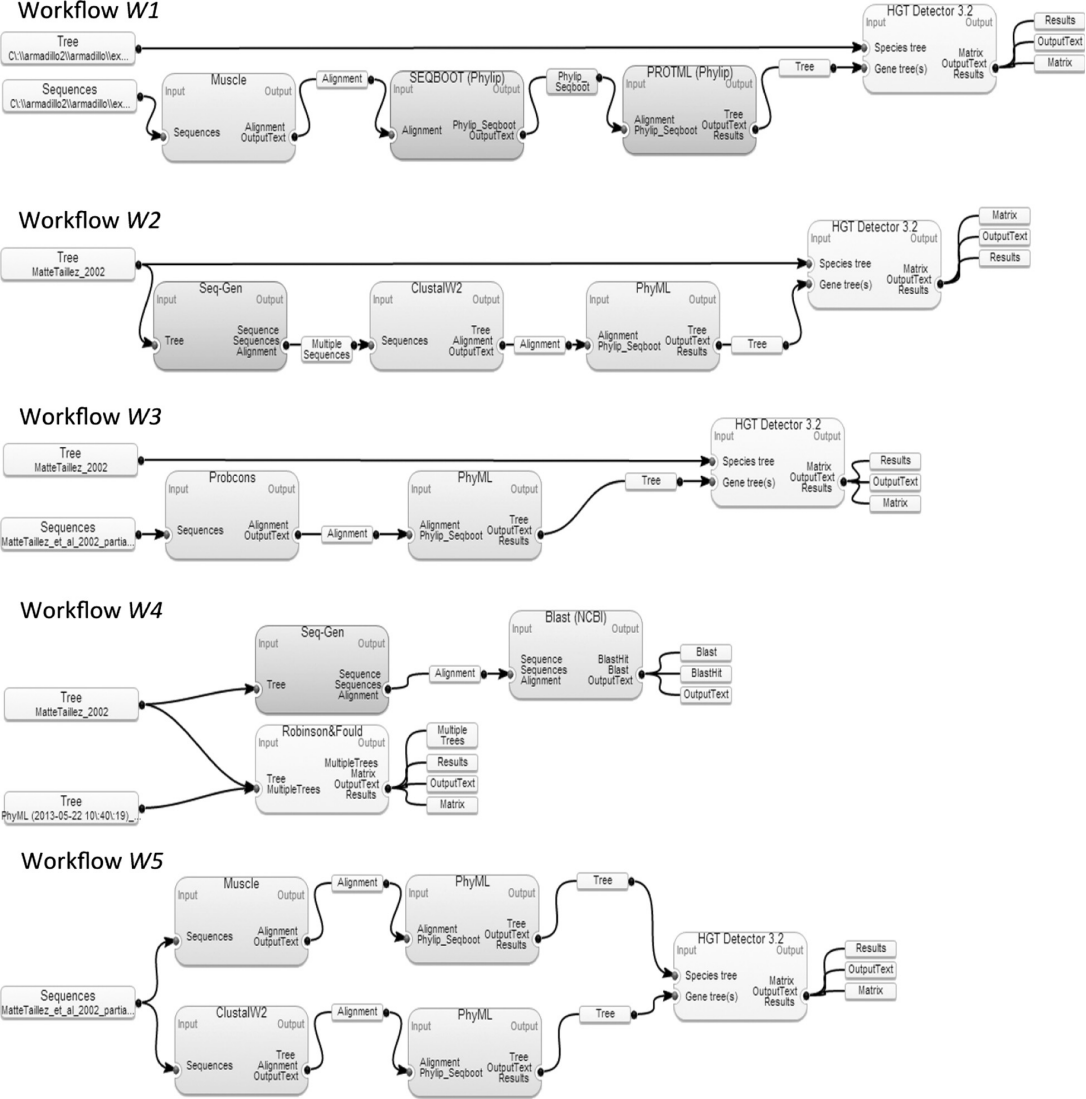
**Five bioinformatics workflows created using the** ***Armadillo*** **WfMS.** These workflows were used to illustrate the workflow encoding strategies discussed in the article. The four workflow encoding types discussed and tested in this study are presented in Table 1.

A typical workflow entails a series of interconnected tasks, the first of which is called an input and the last an output. Such pipelines of tasks can be used to model any sequence of interrelated processes [1]. Workflow complexity can extend from simple execution charts to sophisticated systems allowing for conditional dataflow scheduling and task distribution [2,3]. The primary use of workflows was related to their applications in the business and financial environments [4,5]. Nowadays, workflows are widely applied in many scientific fields, including bioinformatics, for conducting complex scientific analyses as well as for carrying out simulation studies required for testing and validating new statistical methods and software [6]. Scientific workflow management systems (WfMS) created by some research groups have been designed to simplify the workflow generation process, including data refactoring, data processing and results visualization [4,7]. The two best known scientific WfMS dedicated to the field of computational biology are the web-based platform *Galaxy* [2] and the desktop-based platform *Taverna* [4]. These platforms rely on a specific internal programming language and computational model supporting automation. We have recently developed a novel desktop-based bioinformatics WfMS, called *Armadillo* [8], which is primary dedicated to phylogenetic analysis. The *Armadillo* platform, which allows the users to determine execution times of the available tasks, was used here for generating real-life bioinformatics workflows tested in our simulations (see Figure 1 for an example of five bioinformatics workflows created using *Armadillo*).

The most common objectives of workflow clustering consist in discovering, reusing and repurposing existing workflows to achieve a defined goal [9]. One of these goals concerns the optimization of the overall execution time of the given set of workflows by reducing the impact of queue wait times [10]. For instance, Vairavanathan and colleagues [11] have recently developed an optimized workflow file system for cloud computing which, given the structural workflow information, can decrease the workflow execution time by a factor of seven. Furthermore, the problem of identification of similar sub-workflows is central to many tasks scheduling problems [12]. By labeling and dividing large workflows into sub-workflows, Singh and colleagues [13] were able to minimize overall workflow execution time by up to 97%. The latter authors carried out their experiments with large astronomy workflows using the NCSA TeraGrid cluster. Tsai and colleagues [12] reported that an effective use of idle time slots between scheduled tasks is a promising direction for efficient multiple workflow scheduling. They developed a new approach, providing an average execution time gain of 51%, to further improve multiple workflow scheduling performance by clustering certain workflow tasks into groups before the allocation of computational resources. Likewise, Chen *et al.* [14] addressed the problem of merging multiple short tasks into a single one in order to reduce the scheduling overhead and improve the overall runtime performance. The latter authors presented three balancing methods to quantitatively measure workflow characteristics based on task runtime variation, task impact factor, and task distance variance.

In this study, we define and evaluate four workflow encoding schemes which can be used for regrouping workflows either containing similar tasks, or having similar execution times, or using similar keywords (or meta-data), or having similar workflow structures. Note that here we address the problem of clustering the entire workflows without considering the sub-clustering of individual workflow tasks. First, we briefly review the existing works on workflow classification. The partitioning methods used for workflow clustering are discussed afterwards, followed by the description of hierarchical classification methods. We then present four workflow encoding schemes suitable for bioinformatics projects, which were tested in our study using the weighted versions of the *k-*means [15] and *k-*medoids [16] partitioning algorithms in conjunction with three well-known clustering criteria: Calinski-Harabasz [17], logSS [18] and Silhouette [19] indices. The detailed results of hierarchical clustering are presented as well. Finally, a novel cluster stability validation measure is discussed and evaluated in the context of workflow classification, followed by a conclusion section.

### Literature review on workflow clustering

A number of recent studies have addressed the problem of workflow classification [20]. Generation of workflow clusters can be categorized either into language-based approaches or into structure-based approaches [21]. In language-based approaches, string distance measures, such as the Hamming or Levenshtein distances, can be applied to assess dissimilarities between workflows [22]. Language-based methods rely on the text mining of workflow metadata and the use of keyword similarity measures [6,23]. For example, by considering the occurrence matrix of natural language terms found in workflow directories, Costa *et al.* [6] found that in more than 90% of cases the workflow clustering method they proposed was able to partition a coherently set of 53 heterogeneous workflows. The latter authors determined, however, that the considered metadata were sparse and not evenly distributed in the different evaluated workflow formats and repositories. In structure-based workflow clustering, dissimilarities between workflows depend on the adopted workflow graph representation. Graph-based distances, such as the edit, subgraph isomorphism and maximum common induced subgraph distances, have been actively used in this context [21,24]. This means that structure-based workflow clustering methods usually have higher algorithmic complexities [25]. Workflows can also be converted into binary vector representations, where each available workflow task (*i.e.* method, element or activity) is either present (1) or absent (0). If a vector representation is considered, similarity metrics such as the cosine, Euclidean or squared Euclidean distances can be employed to estimate the distances between the observed workflows [20,26]. However, using only the presence-absence data in the workflow representation discards structural information characterizing the dataflow. To circumvent this representation bias, one can apply a multiple vector encoding strategy, such as a transition vector or a process vector encoding [24]. Transition vector encoding strategies were tested by Kastner *et al.* [23] by using several clustering algorithms. Furthermore, Wombacher and Li [21] adopted an N-gram representation of workflows in which adjacent tasks linked together were used to define a specific alphabet. This alphabet was then considered as a base either to encode a vector-like workflow representation or to define an edit distance between workflows.

Some other useful clustering information can be extracted from workflows beside the number or type of tasks, input and output port of tasks and connections between tasks. Statistics such as the average task execution time, the size of transmitted data, the success or failure of each task as well as the selected tasks’ parameters can be also taken into account when clustering workflows [23,27]. For example, Silva *et al.* [27] developed the *SimiFlow* program which accepts as input different workflow formats and takes into account the workflow structure, activity type, input-output ports and relationships between the supplied activities (*e.g.* distance between two activities in the graph) in the workflow clustering process.

## Methods

### Partitioning methods for workflow clustering

The use of partitioning methods for workflow clustering was first considered by Santos *et al.* [20] and Kastner *et al.* [23]. To account for workflow structural information, Santos and colleagues used as workflow similarity measures the maximum common induced subgraph distance as well as the cosine distance between workflow binary vector representations. They then carried out the *k-*means partitioning algorithm for vector-based representation of workflows and the *k-*medoids partitioning algorithm for graph-based representations. Kastner *et al.* [23] encoded the transitions between two separated tasks and used the cosine distance in conjunction with *k*-means in their simulations.

The *k*-means algorithm [15,28] is a partitioning classification algorithm which iteratively regroups into *K* clusters a set of *n* elements (*i.e.* objects, taxa, or workflows in our study) characterized by *m* variables (*i.e.* tasks, or bioinformatics methods in our study), while the cluster centers are chosen to minimize the intra-cluster distances. The most commonly used distances in the framework of *k*-means partitioning are the Euclidean distance, Manhattan distance and Minkowski distance. Each cluster is centered around a point, called the cluster *centroid*, which represents the average coordinate of the cluster’s elements. One of the drawbacks of *k*-means is that this centroid has no real meaning and must be recalculated at each iteration. While a general problem of *k*-means partitioning is *NP*-hard, several proposed polynomial-time heuristics require *O*(*K* × *n* × *m* × *i*) operations to find a clustering solution, where *i* is the number of the algorithm’s iterations.

The *k*-medoids algorithm [16] is a modification of *k*-means in which the centroids, named *medoids*, are representative elements of the cluster. The medoids are chosen at each iteration in order to minimize the intra-cluster distances. The main advantage of this method is that it is more robust than *k*-means in the presence of noise and outliers [29]. The *k*-medoids algorithm has, however, a higher complexity of *O*(*K* × (*n* − *K*)^2^ × *m* × *i*).

In 2001, Makarenkov and Legendre [30] described a weighted version of the *k-*means partitioning algorithm. The following optimization problem was considered when adding weights to the algorithm. Let **W** be a two-way matrix containing measures for *n* elements (*i.e.* workflows; they are represented by the matrix columns) and *m* variables (*i.e.* individual workflow tasks, or pairs of tasks; they are represented by the matrix rows). Let **y** = {*y*_1_, …, *y*_*m*_} be the vector of weights assigned to the variables. In the workflow clustering context the weights can reflect the tasks execution times. Following Makarenkov and Legendre [30], we used Equations 1 and 2 to define the Euclidean distance *d* between workflows and the related optimization problem:

1 $$ {d}_{ij}=\sqrt{{\displaystyle \sum_{p=1}^m{y}_p{\left({w}_{ip}-{w}_{jp}\right)}^2}}. $$

2 $$ {\displaystyle \sum_{k=1}^K\left[{\displaystyle \sum_{i,j=1}^{n_k}{d}_{ij}^2}\right]}/{n}_k\to \min, $$

where *K* denotes the total number of clusters and *n*_*k*_ the number of elements in cluster *k*.

We also consider the cosine distance, which can be represented under the following weighting form (Equation 3):

3 $$ {d}_{ij}=1- \cos \theta =1-\frac{{\displaystyle \sum_{p=1}^m{y}_p\left({w}_{ip}\times {w}_{jp}\right)}}{\sqrt{{\displaystyle \sum_{p=1}^m{y}_p{w}_{ip}^2}}\times \sqrt{{\displaystyle \sum_{p=1}^m{y}_p{w}_{jp}^2}}}. $$

In their pioneering work, Santos *et al.* [20] were first to use the traditional (*i.e.* unweighted) cosine distance in the framework of workflow clustering. This distance is particularly useful in case of sparse binary matrices. One of the disadvantages of the *k*-means and *k*-medoids partitioning algorithms is the need to select the number of clusters prior to performing the clustering. This issue has been rarely addressed in the context of workflow classification [20]. Here, we will carry out the evaluation of the optimal number of clusters using the three following criteria: Calinski-Harabasz [17], logSS [18] and Silhouette [19] indices. We will determine which of them is better suited for classification of bioinformatics workflows under different simulation conditions. The Calinski-Harabasz and logSS indices were considered based on their superior clustering performances as described in Milligan and Cooper [31], while the Silhouette index was selected following the evaluation of Arbelaitz *et al.* [32].

The Calinski-Harabasz (CH) criterion is a ratio-type index considering both the inter-cluster and intra-cluster variances (Equation 4). Here, *SS*_*B*_ is the overall inter-cluster variance, *SS*_*W*_ is the overall intra-cluster variance, *K* the total number of clusters and *n* the number of workflows:

4 $$ CH(K)=\frac{S{S}_B}{S{S}_W}\times \frac{\left(n-K\right)}{\left(K-1\right)}. $$

The *SS*_*B*_ coefficient (Equation 5) is evaluated by calculating the L2 norm (Euclidean distance) between the vectors *mean*_*k*_ (*k* =1 … *K*; *mean*_*k*_ represents the centroid or medoids of cluster *k*) and the vector *mean*, representing the overall mean of the sample data; here, *n*_*k*_ is the number of elements in cluster *k*. The *SS*_*W*_ coefficient (Equation 6) can be calculated in a similar fashion; here, *w*_*ik*_ is the vector representing workflow *i* in cluster *k*. When the Calinski-Harabasz criterion is considered, the number of clusters corresponding to its highest value is selected as the optimal one.

5 $$ S{S}_B={\displaystyle \sum_{k=1}^K{n}_k}{\left\Vert mea{n}_k- mean\right\Vert}^2, $$

and

6 $$ S{S}_W={\displaystyle \sum_{k=1}^K{\displaystyle \sum_{i=1}^{n_k}{\left\Vert {w}_{ik}-mea{n}_k\right\Vert}^2}}. $$

The logSS index (Equation 7) relies on the same inter-cluster and intra-cluster variances to suggest the optimal number of clusters. When the logSS criterion is considered, the optimal number of clusters, *K*, corresponds to the smallest difference between two subsequent logSS scores (logSS(*K*) and logSS(*K* + 1)).

7 $$ \mathrm{logSS}(K)= \log \frac{S{S}_B}{S{S}_W}. $$

On the other side, the Silhouette index estimates how strongly an element belongs to its current cluster rather than to the nearest one. For each workflow *i* in the given workflow set *W* = {*w*_1_, …, *w*_*m*_}, *a*(*i*) denotes the average distance between *i* and all other elements (*i.e.* workflows) in the cluster *c*_*i*_ to which *i* belongs. For any cluster *c*, apart from *c*_*i*_, *d*(*i*, *c*) is defined as the average distance between *i* and all other workflows in *c*. Then, *b*(*i*) represents the smallest of these distances among all such clusters different from *c*_*i*_. The cluster *c*, for which *d*(*i*, *c*) = *b*(*i*) can be considered the neighbor of *i*. Thus, the mean of the Silhouette widths for a given cluster *c*_*k*_ can be computed as follows (Equation 8):

8 $$ s(k)=\left[{\displaystyle \sum_{i=1}^{n_k}\frac{b(i)-a(i)}{ \max \left(a(i),b(i)\right)}}\right]/{n}_k. $$

Using *s*(*k*)’s from Equation 8, the optimal number of clusters *K* is defined as that having the maximum average Silhouette width, $\overline{s}(K)$ (Equation 9):

9 $$ \overline{s}(K)={\displaystyle \sum_{k=1}^K\left[s(k)\right]/K}. $$

### Hierarchical classification methods for workflow clustering

In this study, four different hierarchical classification methods were considered: Unweighted Pair Group Method with Arithmetic Mean (UPGMA) [33], the Neighbor-Joining (NJ) method of Saitou and Nei [34], and the Fitch and Kitsch methods implemented by Felsenstein [35,36]. These hierarchical classification methods can be applied directly to distance matrices calculated using the four encoding schemes discussed below. The UPGMA and Kitsch methods provide an ultrametric classification (*i.e.* ultrametric tree), in which the tree edges cannot be of arbitrary length; they are constrained so that the total length of a unique path from the root of the tree to any tree leave is the same. The NJ and Fitch methods returns a more general tree classification corresponding to an additive, or phylogenetic, tree (*i.e.* the corresponding tree distance satisfies the four-point condition [37]).

The Fitch and Kitsch algorithms rely on the objective least-square function aiming at minimizing the sum of the squared differences between the observed and predicted distances between the elements [37]. Equation 10 describes such a minimization process, where *d*_*ij*_ is the observed distance between elements *i* and *j*, and *δ*_*ij*_ is the estimated tree distance equal to the length of the path between *i* and *j* in the obtained ultrametric or additive tree. The exponent *p* equals 2 in the case of the Fitch and Kitsch algorithm [38]:

10 $$ {\displaystyle \sum_i{\displaystyle \sum_j\frac{{\left({\delta}_{ij}-{d}_{ij}\right)}^2}{d_{ij}^p}}}\to \min . $$

The NJ algorithm follows the principle of minimum evolution, aiming at minimizing the total length of the obtained additive tree, whereas the UPGMA is a simple and widely-used bottom-up agglomerative clustering algorithm. The time complexity of the Fitch and Kitsch algorithms is *O*(*n* ^4^), while that of NJ is *O*(*n* ^3^), and that of UPGMA is *O*(*n* ^2^) for an input dissimilarity matrix of size (*n*x*n*). We used these hierarchical clustering algorithms to compare the four workflow encoding schemes and their different variants presented in the next section.

### Workflow encoding schemes

In this section, we discuss four general types of workflow encoding. The workflows need to be encoded in a matrix form prior to the application of clustering algorithms. In addition, a vector of the tasks weights can be provided to characterize the workflow tasks. The variable weights are often used to indicate the importance of some variables or to reduce the data dimension [30]. For example, the weights can be considered to account for inverse term-frequencies when clustering textual data [26]. Unlike the approach of Makarenkov and Legendre [30], which considers that all weights are non-negative and their sum equals 1, we assume in this study that the weights are user-defined and are only subject to the non-negativity constraint. An example of the four discussed types of workflow encoding is given in Table 1. It concerns the five bioinformatics workflows depicted in Figure 1.

**Table 1:** **The four proposed workflow encoding schemes and their associated weight vectors for the five real-life bioinformatics workflows depicted in Figure** **1**

**Encoding of type I**	***W1***	***W2***	***W3***	***W4***	***W5***	**Weights for encoding of type I**
Blast (NCBI)	0	0	0	1	0	0.35
ClustalW2	0	1	0	0	1	0.49
HGT Detector 3.2	1	1	1	0	1	0.88
Muscle	1	0	0	0	1	0.41
PROTML (Phylip)	1	0	0	0	0	0.68
PhyML (1)	0	1	1	0	1	1.13
PhyML (2)	0	0	0	0	1	1.13
Probcons	0	0	1	0	0	0.55
Robinson & Foulds distance	0	0	0	1	0	0.25
SEQBOOT	1	0	0	0	0	0.14
Seq-Gen	0	1	0	1	0	0.43
**Encoding of type II**	***W1***	***W2***	***W3***	***W4***	***W5***	**Weights for encoding of type II**
Blast (NCBI)	0	0	0	1	0	0.10
ClustalW2	0	1	0	0	1	0.10
HGT Detector 3.2	1	1	1	0	1	1.00
Muscle	1	0	0	0	1	0.10
PROTML (Phylip)	1	0	0	0	0	0.10
PhyML	0	1	1	0	2	0.10
Probcons	0	0	1	0	0	0.10
Robinson&Foulds distance	0	0	0	1	0	0.10
SEQBOOT	1	0	0	0	0	0.10
Seq-Gen	0	1	0	1	0	0.10
**Encoding of type III**	***W1***	***W2***	***W3***	***W4***	***W5***	**Weights for encoding of type III**
Blast (NCBI)	0	0	0	1	0	0.35
HGT Detector 3.2	1	1	1	0	1	0.88
Robinson & Foulds distance	0	0	0	1	0	0.25
ClustalW2 → PhyML	0	1	0	0	1	1.62
Muscle → PhyML	0	0	0	0	1	1.54
Muscle → SEQBOOT (Phylip)	1	0	0	0	0	0.55
PROTML (Phylip) → HGT Detector 3.2	`1	0	0	0	0	1.56
PhyML → HGT Detector 3.2	0	1	1	0	2	2.01
Probcons → PhyML	0	0	1	1	0	1.68
SEQBOOT (Phylip) → PROTML (Phylip	1	0	0	0	0	0.82
Seq-Gen → Blast (NCBI)	0	0	0	1	0	0.78
Seq-Gen → ClustalW2	0	1	0	0	0	0.92
**Encoding of type IV**	***W1***	***W2***	***W3***	***W4***	***W5***	**Weights for encoding of type IV**
Blast (NCBI)	0	0	0	1	0	0.10
HGT Detector 3.2	1	1	1	0	1	1.00
Robinson & Foulds distance	0	0	0	1	0	0.10
ClustalW2 → PhyM	0	1	0	0	1	0.10
Muscle → PhyML	0	0	0	0	1	0.10
Muscle → SEQBOOT (Phylip)	1	0	0	0	0	0.10
PROTML (Phylip) → HGT Detector 3.2	1	0	0	0	0	1.00
PhyML → HGT Detector 3.2	0	1	1	0	2	1.00
Probcons → PhyML	0	0	1	0	0	0.10
SEQBOOT (Phylip) → PROTML (Phylip)	1	0	0	0	0	0.10
Seq-Gen → Blast (NCBI)	0	0	0	1	0	0.10
Seq-Gen → ClustalW2	0	1	0	0	0	0.10
INPUT_Sequences	1	0	1	0	1	1.00
INPUT_Tree	1	1	1	2	0	1.00
OUTPUT_Blast (NCBI)	0	0	0	1	0	1.00
OUTPUT_Matrix	1	1	1	1	1	1.00
OUTPUT_MultipleTrees	0	0	0	1	0	1.00
OUTPUT_OutputText	1	1	1	2	1	1.00
OUTPUT_Results	1	1	1	1	1	1.00

The two instances of the PhyML method used in workflow W5 are indicated as PhyML (1) and PhyML (2) in the encoding of Type 1.

#### Workflow encoding of Type I

The simplest way of workflow encoding is the data presentation in the form of a binary matrix accounting for the *presence and absence* of the available tasks. In the example of the five bioinformatics workflows (Figure 1), the presence and absence of 10 phylogenetic methods encountered in these workflows was first encoded (Table 1). Such an encoding was suggested by many researchers, including Kastner *et al.* [23] and Costa *et al.* [6]. Moreover, as an extension of the work of Costa *et al.* [6], here we use weights representing average execution times of the tasks. The average execution times of the 10 considered phylogenetic methods (for the selected type of input) are indicated in Table 1. This general encoding type can be employed to regroup some similar workflows either to execute them together on a dedicated server or to dispatch some of the lengthy workflows to different servers in order minimize the total execution time of the given workflow set [11,13,14].

#### Workflow encoding of Type II

The workflow encoding of Type II is based on the tasks *occurrence information*. Here we also consider weights for each of the available phylogenetics methods (see Figure 1). These weights can be user-defined and not necessarily related to the tasks execution times. For instance, in the example shown in Table 1 (see encoding of Type II), the method called HGT Detector 3.2 received the weight of 1.0, whereas the nine remaining tasks received the weight of 0.1. The applied weights can be defined by the user through the introduction of specific keywords characterizing certain tasks; the corresponding task’s weight can be given following the presence or absence of these keywords in the method’s annotations. This type of encoding could be particularly useful for searching and selecting the appropriate workflows in a large databank of available workflows characterized by their metadata.

#### Workflow encoding of Type III

To investigate whether the workflow structural information can provide a better workflow classification compared to the presence-absence and occurrence encodings, we represented the five workflows from Figure 1 as connected directed graphs and encoded them into a pair-of-tasks format (see encoding of type III in Table 1). This type of workflow encoding, which is similar to the N-gram encoding of Wombacher and Li [21], preserves the essential structural information without carrying out lengthy graph theory methods aimed at the determining the distance matrix between workflows. The average execution time vector characterizing each available pair-of-tasks is used to define weights in this type of clustering. Vairavanathan *et al.* [11] described a workflow-aware file system which, provided the workflow structural information, allows for a faster execution in cloud computing. The structure-dependent workflow clustering was also discussed by Kastner *et al.* [23].

#### Workflow encoding of Type IV

Finally, we also considered the addition of input and output port information to the pair-of-tasks matrix. This type of encoding, which takes into account the starting and ending points of each workflow, emphasizes the importance of input and output data types. Such an encoding can be particularly useful in situations in which the user can take advantage of the complex workflows which have been already executed with the input and output data similar to those specified by the user. This type of workflows includes lengthy and sophisticated bioinformatics workflows intended for extracting, scanning or processing high-volume genomic data [3]. The weight vector for this type of workflow encoding is defined as follows: the weight of 1 is assigned to the variables encoding input and output ports as well as to the variables associated with the selected tasks (*e.g.* computational methods corresponding to specific keywords); the weight of 0.1 is given to the variables corresponding to the remaining tasks.

Depending on the encoding scheme, the five workflows illustrated in Figure 1 were regrouped into the following optimal subsets of clusters, while using the weighted version of the *k-*means partitioning algorithm and the Calinski-Harabasz optimization criterion. Here, *K* denotes the obtained optimal number of clusters. For encoding of Type I: *K* = 4 - {W1}, {W2}, {W3, W4} and {W5}, encoding of Type II: *K* = 3 - {W1,W3,W5}, {W2} and {W4}, encoding of Type III: *K* = 4 - {W1}, {W2,W4}, {W3} and {W5}, and encoding of Type IV: *K* = 4 - {W1}, {W2, W3}, {W4} and {W5}.

## Results and discussion

### Experimental study for partitioning methods

To evaluate the four workflow encoding schemes defined above, we considered a set of 120 bioinformatics workflows created and executed using the *Armadillo* phylogenetic WfMS [8] as well as 100 workflows created using *Taverna* [4] and extracted from the *my*Experiment workflow repository [39] (Table 2). The *Armadillo* dataset contained four original workflow classes (*K* = 4) and 17 different types of tasks for encodings of Type I and II, 30 different types of tasks for encoding of type III, and 47 different types of tasks for encoding of type IV (see Additional file 1: Table S1A). Each workflow from the *Armadillo* dataset was composed of up to eight tasks chosen from a pool of 17 commonly used bioinformatics applications divided into four classes: (1) Multiple sequence alignment methods: Alignment information, ClustalW2, Baliphy, Muscle, Probcons and Kalign; (2) Phylogenetic tree inference methods: Garly, Neighbor, PhyML, ProtML, Seqboot and ProtPars; (3) Horizontal gene transfer detection and tree comparison methods: HGT Detector, Riata, BLAST, Robinson and Foulds distance, and Random tree; and, finally, (4) A mixed sample that entailed the methods from the three above-mentioned classes. The keyword used for encodings of types II and IV was “HGT” (standing for horizontal gene transfer). Thus, the tasks annotated with the word “HGT” received the weight of 1.0, whereas all the other tasks received the weight of 0.1. The 100 workflows forming the *my*Experiment dataset were retrieved from the *my*Experiment web repository using the keywords “phylogenetics” and “bioinformatics”. Among the extracted workflows, we selected those generated using the Taverna WfMS [4] (versions 1 and 2). Since the experimental execution was not possible for all workflows in this dataset, the approximate running time of each of the 318 available methods was established based on our knowledge.

**Table 2:** **Main characteristics of the real-life workflows from the** ***Armadillo*** **and** ***my*** **Experiment datasets explored in our simulation study**

**Dataset**	**Number of workflows (** ***N*** **)**	**Tasks of types I and II**	**Tasks of type III**	**Tasks of type IV**	**Number of classes (** ***K*** **)**	**Keyword used for encodings of types II and IV**
*Armadillo*	120	17	30	47	4	HGT
*my*Experiment	100	318	345	497	15	BLAST

Classification of these workflows into 15 classes (*K* = 15) was based on workflow metadata accessible via the *my*Experiment website (see Additional file 1: Table S1B). For this dataset, the keyword used for encodings of types II and IV was “BLAST”.

**Figure 2:**
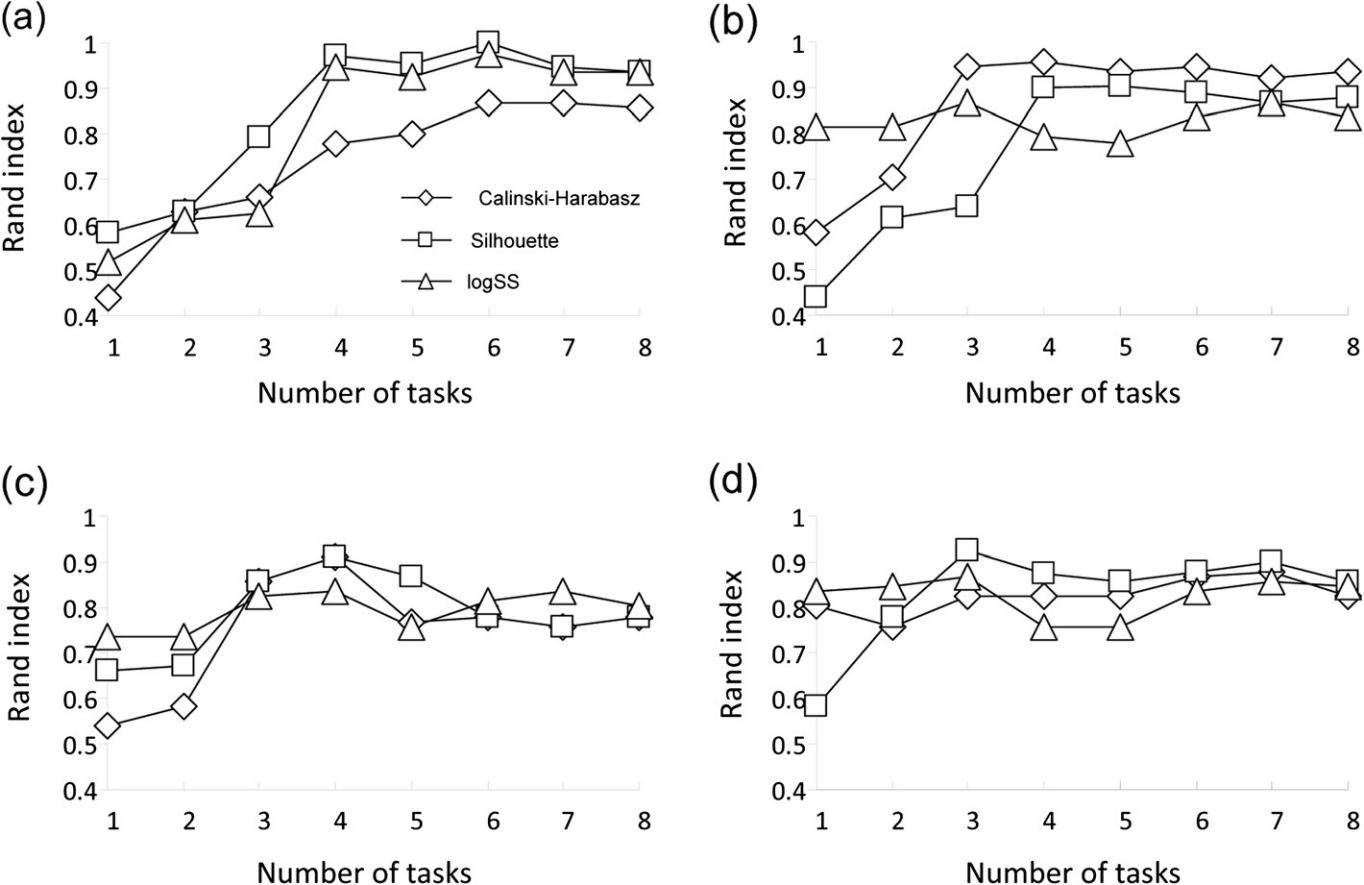
**Simulation results obtained for the four workflow encoding schemes discussed in this article.** The abscissa axis indicates the number of tasks in the workflow; the ordinate axis indicates the corresponding value of the Rand index. The results obtained using encodings of Types I, II, III and IV are represented in panels **(a), (b), (c)** and **(d)**, respectively.

In our first simulation, we considered only the *Armadillo* dataset, the *k*-means partitioning algorithm and the Euclidean distance. For each of the four data encodings discussed in the previous section, the weighted *k*-means algorithm was carried out with an option of 1000 random starts for starting cluster partition and the maximum number of clusters equal to 40. Evaluation of the quality of encoding strategies was done by calculating the Rand index (RI) [40]. The Rand index was calculated by comparing the obtained partition of the set of 120 workflows with the *Armadillo* reference data partition into four classes (see Additional file 1: Table S1A). The Rand index was computed separately for workflows with different numbers of tasks (this number varied from 1 to 8 in the *Armadillo* dataset). Clustering results were presented as a function of the number of methods included in the workflow (Figure 2). The Calinski-Harabasz (CH), Silhouette (SI) and logSS indices were used in turn for determining the optimal number of clusters.

We first evaluated the performances of the basic encoding scheme (Type I, see Figure 2a), consisting of a binary presence-absence matrix accompanied by the weights proportional to the tasks running times. The obtained results suggest that according to the Rand index, SI was superior to the CH and logSS indices for encoding of Type I. The other tendency that can be observed is that the increase in the number of workflow tasks led to the increase in the clustering quality regardless of the selected optimization index (CH, SI or logSS).

Second, we evaluated the encoding scheme of Type II (Figure 2b). The tasks occurrence matrix and the vector of weights corresponding to the keyword “HGT” were considered here. The obtained results suggest that according to the Rand index the CH criterion was superior to SI and logSS for encoding of Type II. The other trend that can be observed is that the increase in the number of the workflow tasks increases the value of RI for all the three optimization criteria.

The third encoding scheme (Figure 2c) consists of the workflow structure representation under the pair-of-tasks format. This type of encoding allows one to take into account structural elements of workflows in contrast to the binary tasks matrices. As in the encoding of Type I, the weights here represented the average execution times of the selected bioinformatics applications. The logSS index here was far from providing the optimal number of clusters in spite of a relatively good performance in terms of RI.

Encoding of Type IV (Figure 2d) puts an emphasis on the input and output types of data. This type of clustering was recommended by Grigori *et al.* [9] and by Wombacher and Li [21]. Unlike the above-mentioned studies, we considered in our encoding only the primary inputs and outputs of workflows, ignoring those of intermediate workflow tasks. This encoding type is in agreement with specifications used in a popular scientific WfMS *Taverna* [4]. We used the weight of 1 for the input and output tasks and for the pairs of tasks related to the HGT Detector method, and the weight of 0.1 for all other available pairs of tasks. Once again, the logSS index was far from providing the optimal number of clusters for this type of data encoding.

The general trend which can be observed in this simulation for all four encoding schemes is that the increase in the number of workflow tasks leads to the increase in the value of RI in the case of the Calinski-Harabasz and Silhouette indices and, in a slighter extent, in the case of logSS.

**Figure 3:**
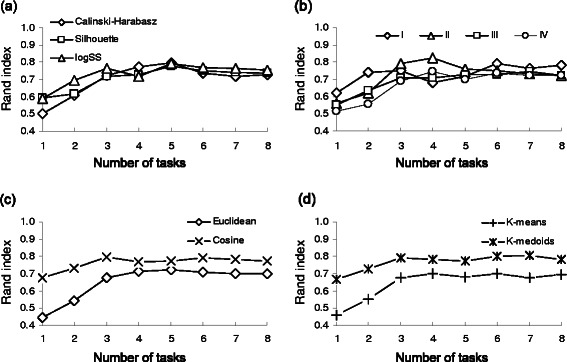
**Average simulation results depicting the behavior of the Rand index as a function of the number of workflow tasks for the** ***Armadillo*** **dataset.** The abscissa axis indicates the number of tasks in the workflow. Panel **(a)** illustrates the effect of the optimization criterion; panel **(b)** - the effect of the encoding type; panel **(c)** - the effect of the distance measure; panel **(d)** - the effect of the applied partitioning algorithm.

**Figure 4:**
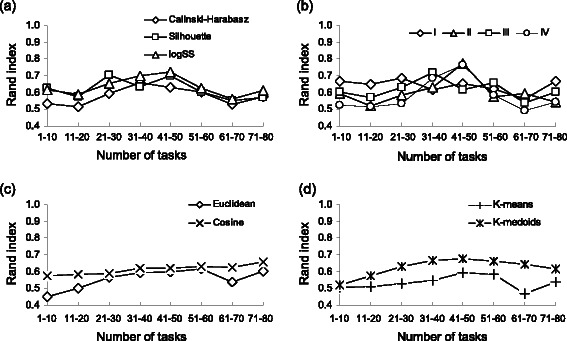
**Average simulation results depicting the behavior of the Rand index as a function of the number of workflow tasks for the** ***my*** **Experiment dataset.** The abscissa axis indicates the interval corresponding to the number of tasks in the workflow; 8 such intervals were considered in our simulation. Panel **(a)** illustrates the effect of the optimization criteria; panel **(b)** - the effect of the encoding type; panel **(c)** - the effect of the distance measure; panel **(d)** - the effect of the applied partitioning algorithm.

**Figure 5:**
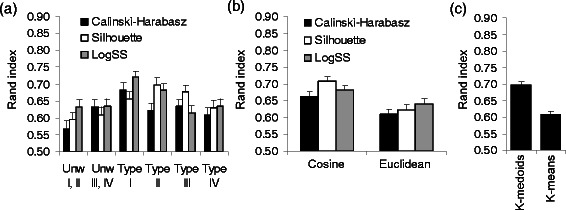
**Combined classification results for the** ***Armadillo*** **and** ***my*** **Experiment datasets obtained using the four encoding types with and without weights (average Rand index ± SEM).** The unweighted encoding strategies were respectively denoted as Unw I,II (combined results for unweighted encodings of Types I and II) and Unw III,IV (combined results for unweighted encodings of Types III and IV). Panel **(a)** illustrates the effect of the optimization criteria for both unweighted (first two sets of bars) and weighted (last four sets of bars) encodings; panel **(b)** - the effect of the distance measure; panel **(c)** - the effect of the applied partitioning algorithm.

Our second simulation was carried out using both the *Armadillo* and *my*Experiment datasets, the weighted *k-*means and *k*-medoids partitioning algorithms and the cosine and Euclidean distances. In this simulation, the options of 100 random starts and of maximum number of clusters equal to 20 were selected. Each data point represented in Figures 3 and 4 is the average taken over all of the parameters combinations except the fixed parameters of interest (*e.g.* in Figure 3a the average is taken over the results obtained by using the *k*-means and *k*-medoids algorithms, the cosine and Euclidean distances and all the four discussed encoding types). Still considering the Rand index as a measure of classification effectiveness, we confirmed that for the *Armadillo* dataset, having more tasks in the workflow generally leads to better classification results regardless of the criterion (CH, Silhouette or logSS) used to select the optimal number of clusters (*p <* 0.01; Figure 3a). The Student-Newman-Keuls test was used here to identify the sample means that were significantly different from each other and the Kolmogorov-Smirnov test to verify the data normality. All statistical tests were carried out using the *InStat* v3.0 program. However, in the simulations conducted with the *my*Experiment dataset (Figure 4a), after a certain point (*i.e.* the 40-50 task interval for this data), having more tasks in the workflow did not result in a better clustering. Such a result can be related to the noise which accumulates with the addition of multiple classification features [30]. Globally, the application of a particular optimization criterion did not have a significant impact on the clustering performance (see Figures 3a, 4a and 5a) in regards to the Rand index (*p >* 0.05).

No significant effect was found when the relation between the workflow encoding type and the number of workflow tasks was considered (Figures 3b and 4b). However, when we combined the results obtained for both of our benchmark datasets (Figure 5a) and considered unweighted encodings, we found significant differences in the average Rand index estimates for encodings of Type I (*p <* 0.01) and Type II (*p <* 0.05), compared to the aggregate unweighted results for these two types of encoding (they are denoted as Unw I,II in Figure 5a). In contrast, no significant difference (*p >* 0.05) was found between the results corresponding to the weighted and unweighted pair-of-tasks matrix encodings (see the diagrams denoted as Unw III,IV, Type III and Type IV in Figure 5a). No significant difference was observed as well when comparing the results obtained using the three considered clustering indices CH, SI and logSS. The SI criterion yielded the best overall Rand index results for encodings of Types II and III, while logSS outperformed the two other clustering indices for encodings of Types I and IV (Figure 5a).

Evaluation of the resulting partitioning as a function of a distance measure, showed that the cosine distance performed significantly better than the Euclidean distance (the average RI of 0.68 vs 0.61, and *p <* 0.001; see Figures 3c, 4c and 5b). The best average clustering results for the cosine distance were obtained regardless of the number of workflow tasks (Figures 3c and 4c). This finding is in accordance with the work of Santos *et al.* [20], who recommended the use of the (unweighted) cosine distance in workflows clustering. Although the Silhouette index provided better average clustering results than CH and logSS when the cosine distance was considered, the obtained difference was not significant. The comparison of the average results returned by the *k*-medoids and *k*-means partitioning algorithms pointed out a significantly better performances of *k*-medoids (average RI 0.70 vs 0.61, *p <* 0.001; see Figure 5c). When the *k*-medoids partitioning was carried out, the SI and logSS indices significantly outperformed the CH index with their respective average RI of 0.71, 0.72 and 0.65, and both *p <* 0.01.

### Experimental study of hierarchical clustering methods

In this section, we discuss the results obtained using the hierarchical clustering methods in the framework of workflow clustering. In this simulation, we tested the four above-defined workflow encoding schemes. Their weighted and unweighted forms were considered. As in our previous simulations, the Euclidean or cosine distances were used to compute distances between workflows from the *Armadillo* and *my*Experiment datasets. The Fitch, Kitsch, Neighbor-Joining (NJ) and UPGMA tree reconstruction algorithms were used to infer hierarchical classifications (*i.e.* additive trees) by running the Fitch, Kitsch and Neighbor programs from the PHYLIP package [36]. Clustering results were evaluated by means of the Robinson and Foulds (RF) topological distance [41] between the obtained trees using the T-Rex website [42]. The resulting trees were compared to the reference trees constructed based on the known workflow classifications (see Additional file 1: Tables S1A and S1B). These reference trees were non-binary as the workflows belonging to the same class were linked together by a multifurcation (a node of degree greater than 3).

**Figure 6:**
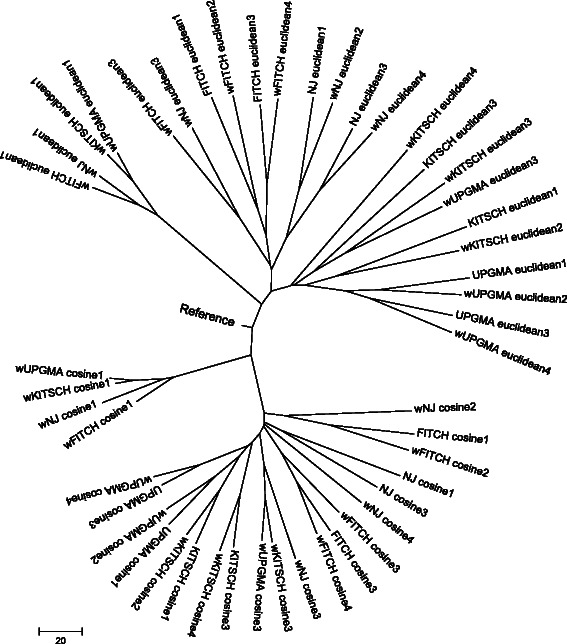
**Classification of hierarchical workflow clustering strategies for the** ***Armadillo*** **dataset (** ***n*** **= 120).** This classification is shown for four weighted and four unweighted workflow encoding types (I, II, III and IV) discussed in this article, the cosine and Euclidean distances and four different hierarchical clustering algorithms (Fitch, Kirsch, NJ and UPGMA). The use of the weighted type of encoding is indicated by the letter “w” preceding the method’s name, while the encoding type is indicated by the corresponding number. The reference taxon represents the optimal tree clustering.

**Figure 7:**
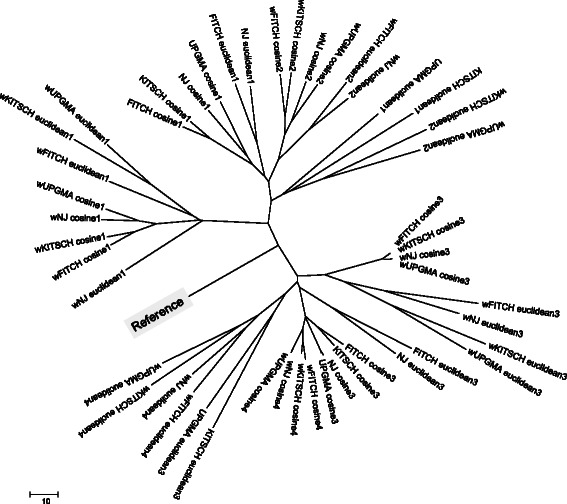
**Classification of hierarchical workflow clustering strategies for the** ***my*** **Experiment dataset (** ***n =*** **100).** This classification is shown for four weighted and four unweighted workflow encoding types (I, II, III and IV) discussed in this article, the cosine and Euclidean distances and four different hierarchical clustering algorithms (Fitch, Kirsch, NJ and UPGMA). The use of the weighted type of encoding is indicated by the letter “w” preceding the method’s name, while the encoding type is indicated by the corresponding number. The reference taxon represents the optimal tree clustering.

As it was impossible to represent each additive tree obtained for each combination of simulation parameters, we decided to compare these trees by using the RF tree distance (to measure topological differences between trees) and the NJ algorithm (NJ was applied to the RF distance matrix) in order to provide a unique hierarchical classification of the obtained trees for both considered experimental datasets (see Figures 6 and 7). In the illustrated classification trees each leave represents an additive tree obtained using the indicated combination of simulation parameters. Visualization of the resulting classifications trees in Figures 6 and 7 was carried out with the program Mega5 [43].

Using the NJ algorithm and the RF distance as a measure of tree proximity, we found that for the *Armadillo* dataset the weighted cosine distance and encoding of Type I provided the best hierarchical clustering when compared to the reference workflow classification. The cluster of four trees obtained using the weighted cosine distance and encoding of Type I is the closest one to the reference tree in terms of the additive distance (*i.e.* the sum of branch lengths of the unique path connecting the related taxa in the tree; see Figure 6). For the *my*Experiment dataset, we found that the weighted cosine and weighted Euclidean distances with encodings of Types I and III provided the best hierarchical classification results (Figure 7).

**Figure 8:**
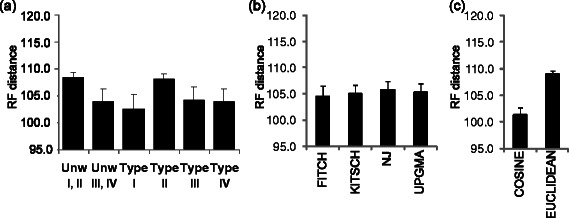
**Combined classification results obtained for the** ***Armadillo*** **and** ***my*** **Experiment datasets (** ***n =*** **220) using hierarchical clustering.** The average Robinson and Foulds topological distance (± SEM) was used to measure clustering performances. The unweighted encoding strategies were respectively denoted as Unw I, II (combined results for unweighted encodings of Types I and II are presented) and as Unw III, IV (combined results for unweighted encodings of Types III and IV are presented). Panel **(a)** illustrates the effect of the encoding type for both unweighted (first two bars) and weighted (last four bars) encodings; panel **(b)** - the effect of the applied hierarchical clustering algorithm; panel **(c)** - the effect of the distance measure.

The aggregated simulation results for both experimental datasets in terms of the average RF distance between the reference trees and the obtained classification trees (Figure 8a) indicate a significant difference between the results corresponding to the unweighted and weighted encodings in the case of encoding of Type I (average RF distance 108.3 vs 102.4; *p <* 0.05). Note smaller values of the RF distance correspond to better clustering results. No significant differences were found for the other types of workflow encoding. When the performances of the four hierarchical clustering algorithms were considered, no significant difference between the corresponding average RF distances was found (Figure 8b). Nevertheless, the Fitch algorithm provided the smallest overall values of RF. Finally, the results yielded by the methods using the cosine and Euclidean distances were also compared (Figure 8c). We found that the use of the cosine distance led to a significantly better cluster recovery in the framework of hierarchical classification (average RF for the cosine distance was 101.3 vs. 109.0 for the Euclidian distance; *p <* 0.001). Summarizing the results obtained for the *Armadillo* and *my*Experiment datasets, we can notice that the best hierarchical classification was found using the Fitch algorithm with the weighted cosine distance and encoding of Type I.

### New pairwise measure of clustering support

**Figure 9:**
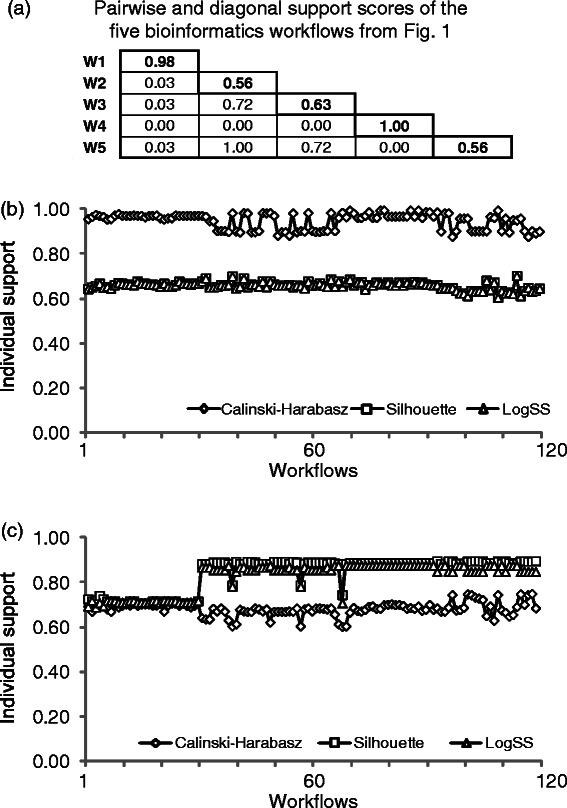
**Simulation results reporting the behavior of the individual pairwise support indices defined in this study.** Panel **(a)** reports the *PS* matrix computed for the set of the five bioinformatics workflows presented in Figure 1 (a support value of 1.0 in the diagonal indicates that the corresponding element was always singleton in its class, whereas a support value of 1.0 in a non-diagonal position indicates that the two corresponding elements were always grouped together); panels **(b)** and **(c)** illustrate the distributions of the global individual *PSG* index obtained for the 120 workflows from the *Armadillo* dataset for the *k-*means and *k-*medoids partitioning algorithms, respectively.

When running our simulations, we could observe some variability in the assignment of individual workflows to their clusters according to the random workflow partition used as a starting point in the *k*-means and *k*-medoids algorithms (Figures 9). For example, a single outlier can influence clustering performances while using these partitioning algorithms [44,45]. In many cases the *k*-means and *k*-medoids heuristics reach only a local minimum which is then returned as a clustering solution [30]. We found that some pairs of workflows were more prone to be assigned to the same class, or to different classes, regardless of the number of classes suggested by the considered clustering index.

Several works have investigated the problem of stability of clustering solution [44-49]. Hennig [44] proposed a method, based on the Jaccard coefficient, for assessing the support of individual clusters of the obtained partitioning solution using a bootstrap resampling. Among different investigated strategies, Hennig recommended the use of Bootstrap, Bootstrap/Jittering or Subsetting together with one of the considered noise generation schemes. In the following work, Hennig [45] described how to estimate the dissolution point and isolation robustness of individual clusters by adding to them new elements or outliers in the framework of the *k*-means or *k*-medoids partitioning. Cheng and Milligan [46] also examined how the addition and removal of elements impacts on the robustness of clustering olutions. On the other hand, Steinley [47] used repeated random restarts of *k*-means to compute a co-occurrence matrix, accounting for pairwise presence-absence of elements in the clustering solution. Moreover, Wang [48] proposed an estimation of the number of clusters by dividing a dataset into two parts and by validating the clustering instability against each of them. Fang and Wang [49] described another bootstrap-based strategy for estimating clustering stability allowing one to select the optimal number of clusters in order to minimize the clustering instability. However, the problem of stability of individual elements has not been addressed so far in the case of partitioning algorithms.

Indeed, a pairwise measure of clustering stability can be introduced in the case when different random starts (*i.e.* different starting partitions) of the partitioning algorithm are considered. Such a measure will reflect the probability of each pair of elements (*i.e.* workflows in our study) to be assigned to the same class or to different classes.

Let *Q* be the number of random starts (*i.e.* iterations, or program runs) of the selected partitioning algorithm (*k*-means or *k*-medoids in our study). Each random start *q* generates a resulting partition, *P*_*q*_, of non-overlapping classes in which each of the *n* considered workflows (each element or object in a general case) is assigned to a certain class. The *pairwise support* score, *PS,* between workflows *w*_*i*_ and *w*_*j*_ can be defined as follows (Equation 11):

11 $$ PS\left({w}_i,{w}_j\right)=\frac{1}{Q}{\displaystyle \sum_{q=1}^Q{S}_{q,ij}}, $$

where *S*_*q*,*ij*_ equals 1 if workflows *w*_*i*_ and *w*_*j*_ are assigned to the same class in the workflow partition *P*_*q*_ obtained at random start *q*, otherwise, it equals 0. The non-diagonal elements of the matrix presented in Figure 9a are the *PS* scores obtained for the five bioinformatics workflows from Figure 1 (the following computational options were used: 100 random starts of *k*-means, CH clustering criterion, cosine distance and encoding of Type I). If a pair of workflows was always assigned to the same class, the corresponding pairwise support is 1 (*e.g.* see the *PS* score for workflows W2 and W5 in Figure 9a).

Then, the *individual (singleton) support score* of each workflow *w*_*i*_, accounting for the probability of *w*_*i*_ to be a singleton element in its class, can be defined as follows (Equation 12; *e.g.* it defines the diagonal elements of the support matrix in Figure 9a):

12 $$ PS\left({w}_i\right)=\frac{1}{Q}{\displaystyle \sum_{q=1}^Q{S}_{qi}}, $$

where *S*_*qi*_ equals 1 if workflow *w*_*i*_ is assigned to a singleton class in the partition *P*_*q*_ obtained at random start *q*, otherwise, it equals 0. For instance, a workflow always classified as a unique element of a singleton class will have the individual support score of 1 and all of the pairwise support scores of 0 (*e.g.* workflow W4 in Figure 9a).

A global clustering solution support measure, *PSG*, for the given set of workflows *W* = {*w*_1_, …, *w*_*n*_} can be defined as follows:

13 $$ PSG(W)=\frac{2\left({\displaystyle \sum_{i=1}^n{\displaystyle \sum_{j=1}^{i-1} \max \left(PS\left({w}_i,{w}_j\right),1-PS\left({w}_i,{w}_j\right)\right)}}+{\displaystyle \sum_{i=1}^n \max \left(PS\left({w}_i\right),1-PS\left({W}_1\right)\right)}\right)}{n^2}. $$

Finally, an individual global workflow support of the workflow *w*_*i*_ (*i* = 1, …, *n*) can be computed as follows:

14 $$ PSG\left({w}_i\right)=\frac{\left({\displaystyle \sum_{j=1\ \left(j\ne i\right)}^n \max \left(PS\left({w}_i,{w}_j\right),1-PS\left({w}_i,{w}_j\right)\right)}\right)+ \max \left(PS\left({w}_i,{w}_j\right),1-PS\left({w}_i\right)\right)}{n}. $$

The first of the two main terms in the numerators of Equations 13 and 14 contains a maximum that accounts for the proportion of times two workflows appear, or do not appear, in the same class over multiple random starts. For instance, two workflows always appearing in the same class or never appearing in the same class contribute the same maximum value of 1, representing maximum possible pairwise clustering stability, to the sum in Equation 14 or to the double sum in Equation 13. The second main term in the numerators of these equations accounts for the stability of the singleton elements. Each equation is then normalized by the total number of individual terms in its numerator. It is worth noting that both the global and individual *PSG* indices vary from 0.5 to 1. The closer the *PSG* index to 1, the higher the robustness of the associated partitioning solution is.

Steinley [47] also considered a measure of pairwise support representing the proportion of times two objects appear in the same group, which is similar to the measure presented in Formula (11). However, Steinley’s work does not discuss any measure accounting for a global support of the obtained clustering solution (Equation 13) or for individual support of the considered objects (Equation 14). The latter work focuses on the recognition of the strongest clustering by permuting the rows of the proportion matrix in order to obtain its block-diagonal form that maximizes the within-block co-occurrences [47].

We investigated how the support measures defined in Equations (11-14) vary with respect to the selected partitioning algorithm, clustering criterion and number of random starts. First, we estimated them for the set of five bioinformatics workflows presented in Figure 1. The overall *PSG* support (Equation 13) for these workflows was found to be 0.90, while the individual global workflow supports (Equation 14) were as follows: *PSG*(W1) = 0.98, *PSG*(W2) = 0.85, *PSG*(W3) = 0.81, *PSG*(W4) = 1.0 and *PSG*(W5) = 0.85. The *k*-means partitioning algorithm, 1000 random starts, CH clustering criterion, cosine distance and encoding of Type I were the selected parameters in these computations.

Second, we considered the *Armadillo* dataset of 120 bioinformatics workflows (see Additional file 1: Table S1A) to evaluate the behaviour of the global and individual *PSG* indices when the *k*-means and *k*-medoids partitioning algorithms were executed with the cosine distance and encoding of Type I (the last two options provided the best average clustering performances in our simulations discussed above). Both partitioning algorithms were tested using 1000 program runs. The distributions of the optimal values of the CH and SI criteria found for 1000 independent runs of the *k*-means and *k*-medoids algorithms are shown in Figures 9b and c, respectively. Table 3 reports the values of the general clustering support index, *PSG*, for the *k*-means and *k*-medoids partitioning algorithms and the CH, SI and logSS clustering indices.

**Table 3:** **General workflow clustering support,** ***PSG*** **(Equation 13), obtained for the** ***Armadillo*** **dataset using as parameters the cosine distance and encoding of Type I**

**Clustering index**	***k*** **-means**	***k*** **-medoids**
Calinski-Harabasz	0.951	0.684
Silhouette	0.659	0.840
logSS	0.659	0.823

Results for the *k*-means and *k*-medoids partitioning algorithms and the CH, SI and logSS clustering criteria are reported. These values were computed over 1000 different program runs for each parameters combination.

We found that in the case of the *k*-means clustering, the CH coefficient produced the highest individual and global scores of workflow support compared to the Silhouette and logSS indices (*i.e. PSG* workflow support of 0.951 for CH vs. 0.659 for both SI and logSS, *p <* 0.0001; see Table 3 and Figure 9b). In the case of the *k*-medoids algorithm, we can observe that the use of CH provided much lower global support values of individual workflow as well as of the global *PSG* index compared to the SI and logSS indices (*i.e. PSG* workflow support of 0.68 for CH vs. 0.84 for SI and 0.82 for logSS, *p <* 0.0001; see Table 3 and Figure 9c). These results are concordant with our simulation findings, where we determined that under the discussed experimental conditions the CH criterion performed better when the *k*-means classification was considered, whereas SI and logSS yielded better results in the framework of the *k*-medoids partitioning.

## Conclusion

In this study, we defined and tested through simulations four workflow encoding schemes combined with specific weighting strategies characteristic for bioinformatics projects. Our findings, based on the analysis of 220 real-life bioinformatics workflows generated by the *Armadillo* [8] and *Taverna* [4] WfMS, suggest that the weighted cosine distance in association with the *k*-medoids partitioning algorithm and the presence-absence workflow encoding provided the highest values of the Rand index among all compared clustering strategies. In our simulations, the Silhouette (SI) and logSS optimization criteria generally outperformed the Calinski-Harabasz (CH) criterion in the framework of *k*-medoids clustering, whereas the CH index generated better classification results in the case of *k*-means clustering. The SI index yielded very steady classification results when used in conjunction with the weighted cosine distance. Our analysis also shows that the application of weights can have a major impact on the clustering solution obtained by partitioning or hierarchical classification algorithms. Overall, the consideration of weight vectors representing either the average execution times of the tasks or the selected keywords allowed us to improve clustering results. As we also illustrated, encodings of Types I and II, based on the presence-absence and occurrence information, generally outperformed more sophisticated encodings of Types III and IV, taking into account structural workflow information and formats of input and output ports. This is mainly due to a greater sparseness of data corresponding to encodings of Types III and IV. The latter conclusion is in accordance with the findings of Wombacher and Li [21], who argued that the N-gram encoding, including the workflow structure information, does not improve the quality of workflow clustering. This is also in accordance with the work of Santos *et al.* [20], who found that workflow task connectivity information does not necessarily bring an additional advantage to the workflow clustering process.

Workflow classification performed using hierarchical methods also favored encoding of Type I in association with the weighted cosine distance. In the future, it would be interesting to compare hierarchical workflow classifications obtained by means of distance methods with those built by means of the maximum parsimony (MP) and maximum-likelihood (ML) approaches. The main advantage of the MP and ML methods is that they can be applied directly to the two-way object-variable matrices without averaging the results through calculating distances between the objects. Moreover, the bootstrap support of the additive trees inferred by the latter methods could be calculated as well.

Furthermore, we also introduced and tested through simulations a novel pairwise measure of clustering solution stability, *PS*, which can be applied in situations when a series of independent program runs is carried out (*e.g.* when different random seeds are used as input of a partitioning algorithm). Such a measure evaluated over multiple random starts reflects the probability of each pair of elements to be assigned to the same class. In addition, we also introduced the global pairwise support index, *PSG*, allowing one to estimate the global support of the proposed clustering solution as well as the global support of individual elements (*i.e.* workflows in our case). In this study, we considered workflows from the field of bioinformatics. It would be important to investigate the presented encoding schemes and the introduced *PS* and *PSG* indices using workflows from other domains, such as economics, business and medicine, as they may have different structural and computational properties.

## Additional file

Additional file 1: Table S1A. The *Armadillo* dataset of 120 workflows and their respective classes. **Table S1B.** The *my*Experiment dataset of 100 workflows (generated using the *Taverna* workflow platform) and their respective classes. Here each workflow is represented by a series of tasks (defined by the users); 2 each of these tasks includes multiple computational methods (not indicated here).
