# Supplementary material for: Classification of bioinformatics workflows using weighted versions of partitioning and hierarchical clustering algorithms
Source: BMC Bioinformatics. 2015 Mar 3;16:68. doi: 10.1186/s12859-015-0508-1 (PMC4354763; doi:10.1186/s12859-015-0508-1)
Supplement: Additional file 1: Table S1A. — The Armadillo dataset of 120 workflows and their respective classes. Table S1B. The myExperiment dataset of 100 workflows (generated using the Taverna workflow platform) and their respective classes. Here each workflow is represented by a series of tasks (defined by the users); 2 each of these tasks includes multiple computational methods (not indicated here). [file 12859_2015_508_MOESM1_ESM.doc]

**Additional file**

**Table S1A – The *Armadillo* dataset of 120 workflows and their respective classes.**

**Table S1A (continued)**

**Table S1B – The *my*Experiment dataset of 100 workflows (generated using the *Taverna* workflow platform) and their respective classes. Here each workflow is represented by a series of tasks (defined by the users); each of these tasks includes multiple computational methods (not indicated here).**

**Table S1B (continued)**
